# A phase 1 study of PARP-inhibitor ABT-767 in advanced solid tumors with BRCA1/2 mutations and high-grade serous ovarian, fallopian tube, or primary peritoneal cancer

**DOI:** 10.1007/s10637-017-0551-z

**Published:** 2018-01-08

**Authors:** Diane A. J. van der Biessen, Jourik A. Gietema, Maja J. A. de Jonge, Ingrid M. E. Desar, Martha W. den Hollander, Matthew Dudley, Martin Dunbar, Robert Hetman, Camille Serpenti, Hao Xiong, Rajendar K. Mittapalli, Kirsten M. Timms, Peter Ansell, Christine K. Ratajczak, Stacie Peacock Shepherd, Carla M. L. van Herpen

**Affiliations:** 1 Erasmus MC Cancer Institute, Erasmus University Medical Center, Rotterdam, The Netherlands; 2 University Medical Center Groningen, University of Groningen, Groningen, The Netherlands; 3 Radboud University Medical Center, Nijmegen, The Netherlands; 4 AbbVie Inc., North Chicago, IL USA; 5 AbbVie B.V, Hoofddorp, The Netherlands; 6 Myriad Genetics Inc., Salt Lake City, UT USA

**Keywords:** PARP inhibitor, BRCA, Solid tumor, Ovarian cancer, Homologous recombination deficiency

## Abstract

*Purpose* This phase 1 study examined safety, pharmacokinetics (PK), and efficacy of the poly(ADP-ribose) polymerase (PARP) inhibitor ABT-767 in patients with advanced solid tumors and *BRCA1/2* mutations or with high-grade serous ovarian, fallopian tube, or primary peritoneal cancer. *Methods* Patients received ABT-767 monotherapy orally until disease progression or unacceptable toxicity. Dose was escalated from 20 mg once daily to 500 mg twice daily (BID). Dose-limiting toxicities, recommended phase 2 dose (RP2D), food effect, objective response rate, and biomarkers predicting response were determined. *Results* Ninety-three patients were treated with ABT-767; 80 had a primary diagnosis of ovarian cancer. ABT-767 demonstrated dose-proportional PK up to 500 mg BID and half-life of ~2 h. Food had no effect on ABT-767 bioavailability. Most common grade 3/4 treatment-related adverse events were nausea, fatigue, decreased appetite, and anemia. Anemia showed dose-dependent increase. RP2D was 400 mg BID. Objective response rate by RECIST 1.1 was 21% (17/80) in all evaluable patients and 20% (14/71) in evaluable patients with ovarian cancer. Response rate by RECIST 1.1 and/or CA-125 was 30% (24/80) in patients with ovarian cancer. Mutations in *BRCA1 or BRCA2*, homologous recombination deficiency (HRD), and platinum sensitivity were associated with tumor response. Median progression-free survival was longer for HRD positive (6.7 months) versus HRD negative patients (1.8 months) with ovarian cancer. *Conclusions* ABT-767 had an acceptable safety profile up to the established RP2D of 400 mg BID and dose-proportional PK. Patients with *BRCA1 or BRCA2* mutation, HRD positivity, and platinum sensitivity were more sensitive to ABT-767.

**Electronic supplementary material:**

The online version of this article (10.1007/s10637-017-0551-z) contains supplementary material, which is available to authorized users.

## Introduction

Poly(ADP-ribose) polymerase-1 (PARP-1) and PARP-2 are nuclear enzymes that recognize DNA damage and facilitate DNA repair [1, 2]. Malignancies with deficiencies in homologous recombination, such as those with breast cancer gene (*BRCA)* mutations, are more dependent on PARP for DNA repair than normal cells and are therefore more sensitive to PARP inhibition [3]. Accordingly, monotherapy PARP inhibitors have shown antitumor activity in *BRCA* mutated tumors [4–8].

In patients with breast cancer, mutations in the *BRCA1/2* genes account for 5% of all breast cancers and 15–20% of all hereditary breast cancers [9, 10]. *BRCA1/2* mutations also account for an increased risk of early-onset prostate cancer, gastric and pancreatic cancer [11]. Approximately 20% of high-grade serous ovarian cancers (HGSOC) have a germline or somatic *BRCA1/2* mutation, and approximately 50% overall have a defect in homologous recombination [12]. The standard treatment for ovarian cancer is surgical debulking and chemotherapy; however, many patients develop resistance to platinum-based chemotherapy after the first or subsequent treatment cycles [13].

ABT-767 is a potent, oral, competitive inhibitor of PARP-1 (Ki = 0.47 nM) and PARP-2 (Ki = 0.85 nM). This compound has shown single-agent anti-tumor activity in patients with HGSOC and *BRCA*-mutated solid tumors [14]. Here, we evaluated the safety/tolerability, pharmacokinetics (PK), food effect, and efficacy of ABT-767 in patients with advanced solid tumors with *BRCA1/2* mutations, and in patients with HGSOC, fallopian tube, or primary peritoneal cancer.

## Materials and methods

### Patients

Patients were screened at three sites in the Netherlands. Eligible patients were 18 years or older with histologically or cytologically confirmed malignancy that was metastatic or unresectable, and for which standard curative measures did not exist or were no longer effective. All patients had either a documented deleterious *BRCA1* or *BRCA2* mutation or high-grade serous ovarian, fallopian tube, or peritoneal cancer, Eastern Cooperative Oncology Group (ECOG) performance status of 0 to 2, and adequate hematologic, renal and hepatic function. In the Expanded Safety Cohort #1, all patients had a documented deleterious *BRCA1/2* mutation, a lesion accessible for biopsy, and measurable disease per Response Evaluation Criteria in Solid Tumors (RECIST), version 1.1. In the Expanded Safety Cohort #2, all patients had a known positive or negative status for deleterious *BRCA1/2* mutation. Patients in the Expanded Safety Cohort #2 with ovarian cancer could have non-measurable disease in case of an elevated serum cancer antigen-125 (CA-125) level by Gynecologic Cancer Intergroup (GCIG) criteria.

Patients were not eligible if they received anti-cancer therapy within 28 days or 5 half-lives (whichever was shorter) of first dose of study drug, if they had central nervous system metastases, unresolved clinically significant toxicities from their prior anti-cancer therapy, clinically significant uncontrolled condition(s), or if they were pregnant or breastfeeding. In the Expanded Safety Cohorts, patients were not eligible if they had received a prior PARP inhibitor.

### Study design and treatment

This was a phase 1, open-label, non-randomized, dose-escalation study (NCT01339650) of ABT-767 to determine the dose-limiting toxicities (DLTs), maximum tolerated dose (MTD) and the recommended phase 2 dose (RP2D). ABT-767 was administered orally to patients on days 1–28 of 28-day cycles. Patients continued to receive ABT-767 until they experienced progression per RECIST 1.1 or unacceptable toxicity. Intra-patient dose escalation was allowed in patients who experienced clinical worsening or who had stable disease and who may benefit from dose escalation in the opinion of the investigator.

Patient cohorts were administered ascending doses of ABT-767. The initial dose was 20 mg once daily (QD). Doses for subsequent cohorts were administered twice daily (BID) and were doubled until a grade 2 toxicity occurred during cycle 1; following a grade 2 toxicity, dose escalations were restricted to between 25% and 75% of the previous dose. The decision to escalate the dose was based on observed DLTs, other adverse events, and PK data. A modified 3 + 3 design was used to determine MTD and RP2D. Each dose level included at least 3 evaluable patients but could enroll up to 9 patients. If one patient within any dose level experienced a DLT, the cohort was expanded to at least 6 patients. The dose could be escalated if >67% of patients in a cohort did not experience a DLT in Cycle 1. MTD was defined as the highest dose level at which less than 2 out of 6 patients or <33% of patients experienced a DLT. The RP2D was defined by observed DLTs and determination of MTD.

After determination of RP2D, additional patients were enrolled to two Expanded Safety Cohorts to further evaluate the safety, tolerability, and PK of ABT-767 at the RP2D. Food effect was assessed in the Expanded Safety Cohort enrolling patients with *BRCA1/2* germline mutation and advanced solid tumors only.

### Safety and tolerability

Safety was evaluated throughout the study through assessment of treatment-emergent adverse events (TEAEs) and laboratory tests. TEAEs were reported according to the National Cancer Institute Common Terminology Criteria for Adverse Events (CTCAE) version 4.03. Treatment-related TEAEs were those considered possibly or probably related to ABT-767.

The following TEAEs were considered DLTs if occurring during the first cycle of dosing and attributed to ABT-767: grade 4 absolute neutrophil count (ANC), grade 3 ANC lasting more than 7 days, or ≥ grade 3 ANC with fever; ≥ grade 3 thrombocytopenia; ≥ grade 3 decreased hemoglobin; non- hematologic toxicities of CTCAE ≥ grade 3 that have increased at least 2 grade levels from baseline (except nausea, vomiting, diarrhea, and tumor pain that have not received optimal treatment); creatinine increases to grade 3 that are not corrected to grade 1 or baseline within 24 h by IV fluids; ≥ grade 3 metabolic toxicities not corrected to ≤ grade 2 within 24 h or any symptomatic grade 4 metabolic toxicity; or grade 2 non-hematologic toxicities representing ≥2 grade increase from baseline requiring dose modification or delay of >1 week.

### Pharmacokinetics

ABT-767 was administered as a single dose under fasting conditions on day −4 (for patients being evaluated for food effect) and as either QD or BID under non-fasting conditions on study days 1 through 28. ABT-767 PK samples were collected at 0, 0.5, 1, 1.5, 2, 4, 6, 8, 10 and 24 h post-dose on Cycle 1 Days −4, 1, and 8. Urine sample collections started immediately after the ABT-767 morning dose on Cycle 1 Day 7 and ended immediately prior to the morning dose on Cycle 1 Day 8. Maximum observed plasma concentration (C_max_), the time to C_max_ (peak time, T_max_), and the area under the concentration curve (AUC_t_) were determined using non-compartmental methods.

### Exploratory efficacy

Objective response rate (ORR: confirmed complete response [CR] plus partial response [PR]) was based on RECIST version 1.1, and was evaluated in patients with measurable disease at baseline. Tumor marker CA-125 response was measured by GCIG criteria [15] in patients with ovarian cancer, and was evaluated in patients with a pre-treatment sample within 2 weeks of starting treatment that was at least twice the upper limit of normal. Time of progression-free survival was defined as the number of days from first dose of study drug to disease progression or death if disease progression was not reached. Six-month progression-free survival (PFS) rate was calculated.

### Biomarker analysis

*BRCA* status was collected at screening if known. A known *BRCA* status was required for patients in the expansion cohorts. Tumor *BRCA1/2* mutation status and homologous recombination deficiency (HRD) score were analyzed using a next generation sequencing assay (Myriad) in patients providing tissue samples in a central lab [16]. Tumors were considered HRD positive if they had an HRD score ≥ 42 and/or a *BRCA1/2* mutation, as previously described [17].

### Statistical analysis

All patients who received at least one dose of ABT-767 were included in the safety, PK, and efficacy analyses. For all statistical analyses, unless otherwise stated, statistical significance was determined using a two-sided *p* value ≤0.05. The Kaplan-Meier method was used to estimate PFS. Data were analyzed both by specific ABT-767 dose cohort and in some cases by pooling multiple cohorts.

Dose, *BRCA* mutation status, platinum sensitivity, baseline CA-125 level (if relevant), and age were examined as potential predictive variables for efficacy (PFS and best tumor response) and safety (anemia). A logistic regression analysis was performed to characterize the dose-response relationship between the ABT-767 dose and best tumor response (CR or PR).

## Results

### Patient characteristics and treatment exposure

A total of 93 patients were enrolled and treated in the dose escalation (*n* = 63) or expanded safety (*n* = 30) cohorts. Patient demographics and baseline clinical characteristics are summarized in Table 1. The majority of patients (86%) had a primary diagnosis of ovarian cancer, and 45% (42/93 patients) had known germline *BRCA1/2* mutations.

**Table 1:** Patient demographic and baseline clinical characteristics

Variable	Dose escalation (*N* = 63)	Expanded safety (*N* = 30)	Total (*N* = 93)
Sex, n (%)			
Female	62 (98)	30 (100)	92 (99)
Age, years			
Mean (SD)	57 (11)	59 (10)	58 (11)
Median (range)	57 (27–80)	60 (33–73)	58 (27–80)
Race, n (%)			
White	63 (100)	28 (93)	91 (98)
Asian	0	2 (7)	2 (2)
Primary diagnosis^a^, n (%)			
Ovarian, fallopian tube, or primary peritoneal	54 (86)	26 (87)	80 (86)
Fallopian tube, n	3	0	3
Primary peritoneal, n	2	1	3
Breast	7 (11)	3 (10)	10 (11)
Pancreatic	0	1 (3)	1 (1)
Prostate	1 (2)	0	1 (1)
Peritoneal mesothelioma	1 (2)	0	1 (1)
Prior therapies, n (%)			
Number of prior therapies			
1	9 (14)	4 (13)	13 (14)
2	17 (27)	11 (37)	28 (30)
3	11 (17)	5 (17)	16 (17)
4	15 (24)	8 (27)	23 (25)
≥ 5	11 (17)	2 (7)	13 (14)
≥ 1 PARP inhibitor-containing therapy	5 (8)	0	5 (5)
≥ 1 platinum-containing therapy	59 (94)	27 (90)	86 (93)
Platinum-free interval < 6 months^b^	32 (51)	10 (33)	42 (45)
Platinum-free interval 6–12 months	20 (32)	11 (37)	31 (33)
Platinum-free interval > 12 months	6 (10)	4 (13)	10 (11)
*BRCA* status, n (%)^c^			
Germline *BRCA1/BRCA2* mutation positive	26 (41)	16 (53)	42 (45)
Germline *BRCA1/BRCA2* mutation negative	11 (18)	13 (43)	24 (26)
Germline *BRCA1/BRCA2* mutation status unknown	26 (41)	1 (3)	27 (29

Abbreviations: *PARP* poly(ADP-ribose) polymerase; *SD* standard deviation

^a^21 patients had a history of other malignancies including breast, colorectal, melanoma, renal, and basal or squamous cell skin cancer

^b^Platinum-free interval was defined as the time in months between last dose of platinum-based therapy and start of the next line of therapy. Platinum-free interval data are missing for 3 patients with prior platinum (1 in Dose Escalation Cohort, and 2 in Expanded Safety Cohort). Patients with a platinum-free interval of <6, 6–12, and >12 months were considered platinum resistant, partially platinum sensitive, and platinum sensitive, respectively

^c^*BRCA1/2* mutation as reported by site at screening

The median duration of ABT-767 treatment among all 93 patients was 3.8 months (range 0.03–31.1) as of data cutoff on March 29, 2016. The median duration for patients in the dose escalation was 3.8 months (range 0.03–20.6), and the median duration for patients in the expanded safety cohorts was 4.0 months (0.5–31.1).

### Dose-limiting toxicities and recommended dose

DLTs occurred in three patients during the DLT evaluation period; angina pectoris in one patient at 20 mg BID, and grade 3 anemia in two patients at 400 mg and 500 mg BID. The RP2D was determined to be 400 mg BID. The 500 mg BID dose was considered intolerable due to grade 3 anemia and fatigue/general malaise.

### Safety

Eighty-seven patients (93.5%) experienced at least one treatment-related adverse event, and 40 patients (43%) experienced at least one grade 3 or 4 treatment-related TEAE. Grade 3 or 4 treatment-related adverse events occurring in more than one patient overall were anemia (31.2%), fatigue (5.4%), decreased appetite (2.2%), neutropenia (2.2%), and thrombocytopenia (2.2%) (Table 2). A dose-dependent increase in all-grade anemia was observed with ABT-767 from 20 mg BID (16.7%) to 500 mg BID (66.7%). Mean hemoglobin levels for all patients from screening visit to Cycle 3 Day 1 are shown in Supplemental Fig. 1.

**Table 2:** Treatment-related adverse events by frequency of grade 3 or 4 events

Event, n (%)	Dose Escalation (N = 63)		Expanded Safety (N = 30)		Total (N = 93)	
	All Grades	Grade 3 or 4	All Grades	Grade 3 or 4	All Grades	Grade 3 or 4
Anemia	17 (27)	17 (27)	14 (47)	12 (40)	31 (33)	29 (31)
Fatigue	34 (54)	3 (5)	18 (60)	2 (7)	52 (56)	5 (5)
Decreased appetite	31 (49)	0	13 (43)	2 (7)	44 (47)	2 (2)
Neutropenia	1 (2)	1 (2)	2 (7)	1 (3)	3 (3)	2 (2)
Thrombocytopenia	2 (3)	2 (3)	0	0	2 (2)	2 (2)
Nausea	34 (54)	0	19 (63)	1 (3)	53 (57)	1 (1)
Leukopenia	0	0	2 (7)	1 (3)	2 (2)	1 (1)

**Fig. 1:**
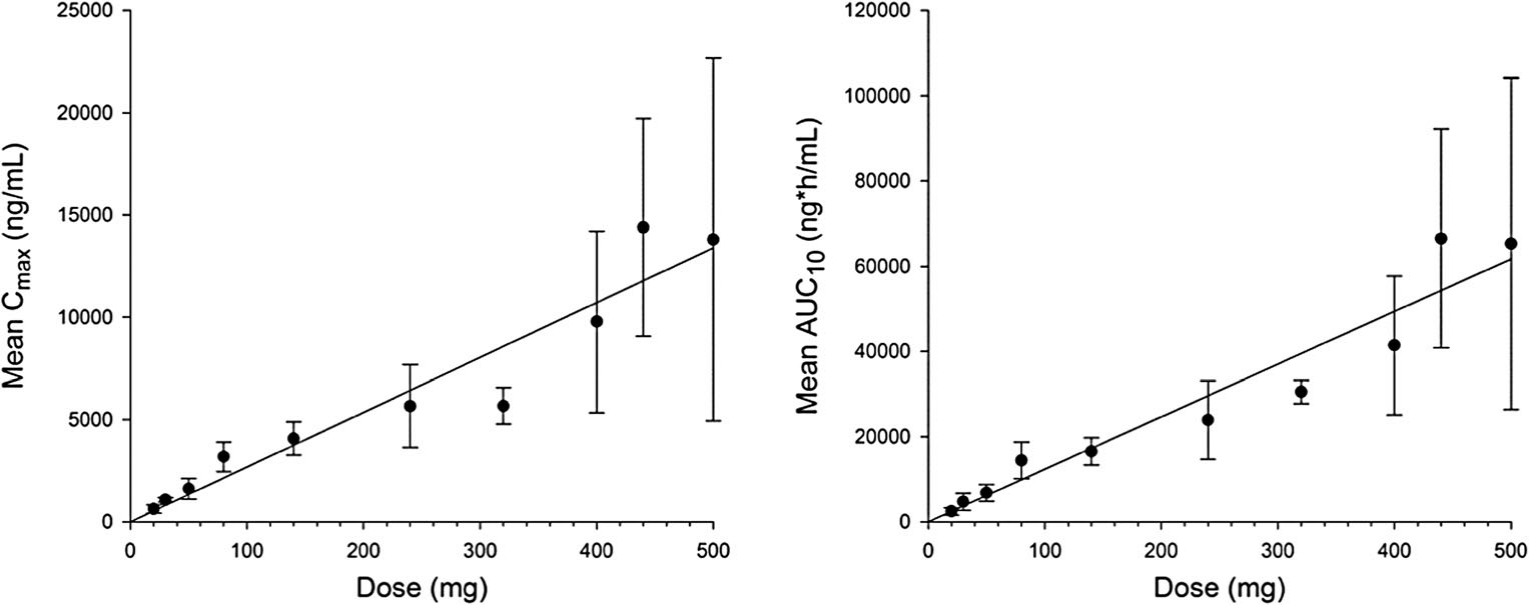
Mean ± SD C_max_ and AUC_10_ after the morning dose of ABT-767 on Day 1 of Cycle 1. Abbreviations: *C*_*max*_ maximum observed plasma concentration; *AUC*_*10*_ area under the plasma concentration–time curve from time 0 to 10 h

Two patients had treatment-related TEAEs that led to discontinuation (thrombocytopenia in one patient at 20 mg BID, and decreased platelet count and anemia in one patient at 400 mg BID). Twenty-nine patients (31.2%) experienced at least one TEAE that led to ABT-767 dose reduction; dose reduction was due to anemia in 20 of these patients. Thirty-five patients had a treatment-related TEAE that led to ABT-767 interruption. Treatment-related TEAEs leading to dose reduction and interruption were generally more frequent with increasing dose.

Six patients (6.5%) experienced at least one treatment-related serious TEAE (dizziness and angina pectoris in one patient; decreased appetite, dehydration, and nausea in one patient; abdominal pain, nausea, malaise, and vomiting in one patient; and malaise, macular hole, and lung infection in one patient each).

### Pharmacokinetics

ABT-767 exposure increased approximately dose-proportionally from 20 mg to 500 mg (Fig. 1). The median T_max_ values ranged from 1.5 to 2.0 h under non-fasting condition, and the harmonic mean half-life was approximately 2 h across different cohorts (Supplemental Table 1). On average, 10% of ABT-767 dose was recovered as the parent drug in urine, and renal clearance appeared to be independent of dose, which suggests that renal clearance plays an important role in ABT-767 elimination. The effect of food on the oral bioavailability of ABT-767 was evaluated up to 400 mg ABT-767 dose, and no significant food effect was seen on C_max_ or AUC of ABT-767.

### Efficacy

**Fig. 2:**
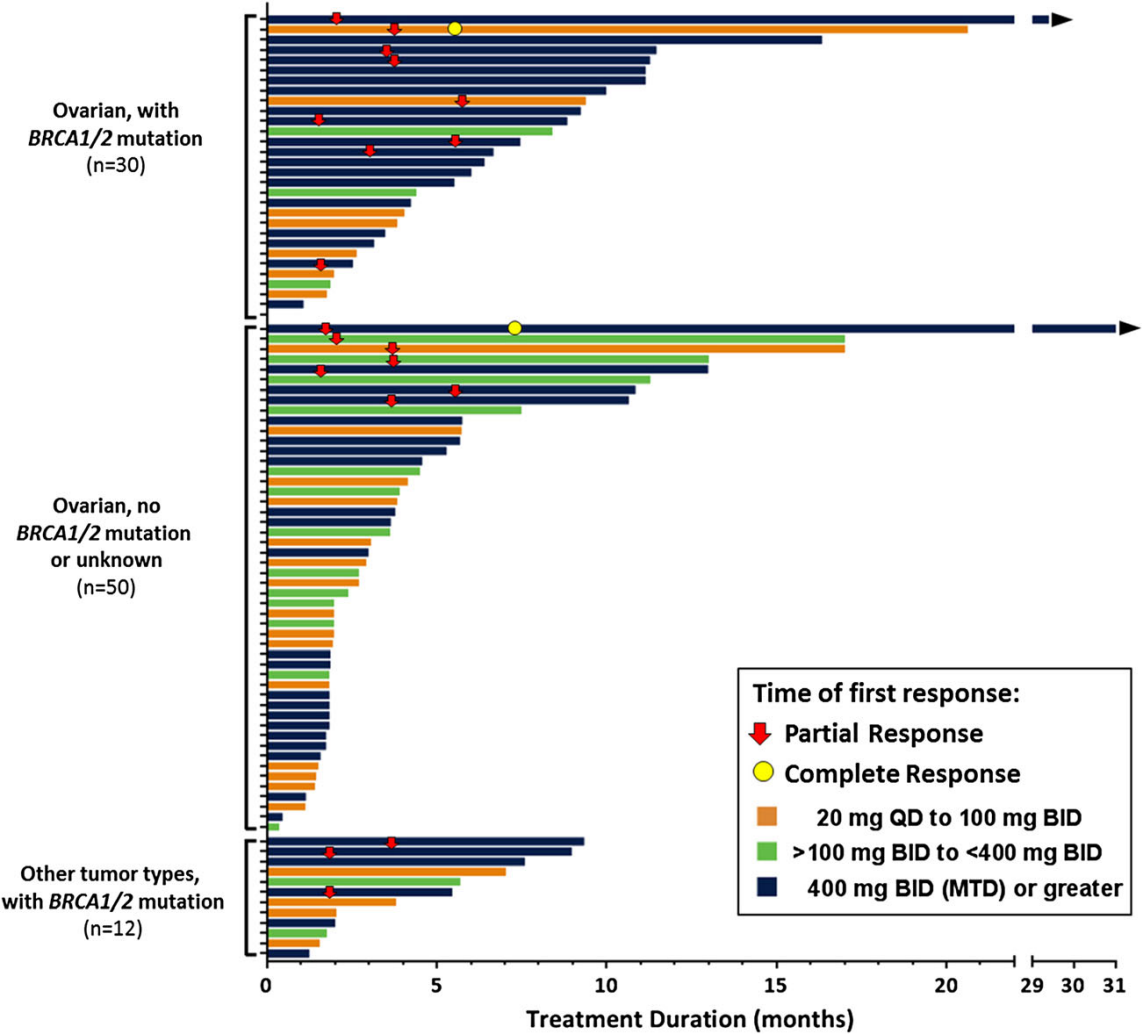
Efficacy data for individual patients. Abbreviations: *BID* twice daily; *MTD* maximum tolerated dose; *QD* once daily. Germline *BRCA* status was provided by the investigators. Responses shown are best tumor responses (RECIST1.1). Arrowhead indicates patients still on study. This plot does not include one patient with peritoneal mesothelioma and no *BRCA1/2* mutation from the 50 mg BID cohort whose best response was stable disease at 15 months

Among all patients, the objective response rate (CR + PR) by RECIST 1.1 criteria was 21% ([17/80], 95% CI: 13–32%). Among patients with ovarian cancer, the objective response rate by RECIST 1.1 criteria was 20% ([14/71], 95% CI: 11–31%), by GCIC (CA-125) criteria 35% ([23/35], 95% CI: 24–48%), and by using RECIST 1.1 and/or CA-125 criteria 30% ([24/80], 95% CI: 20–41%). Duration of therapy and best tumor response (RECIST 1.1) for individual patients are shown in Fig. 2.

**Fig. 3:**
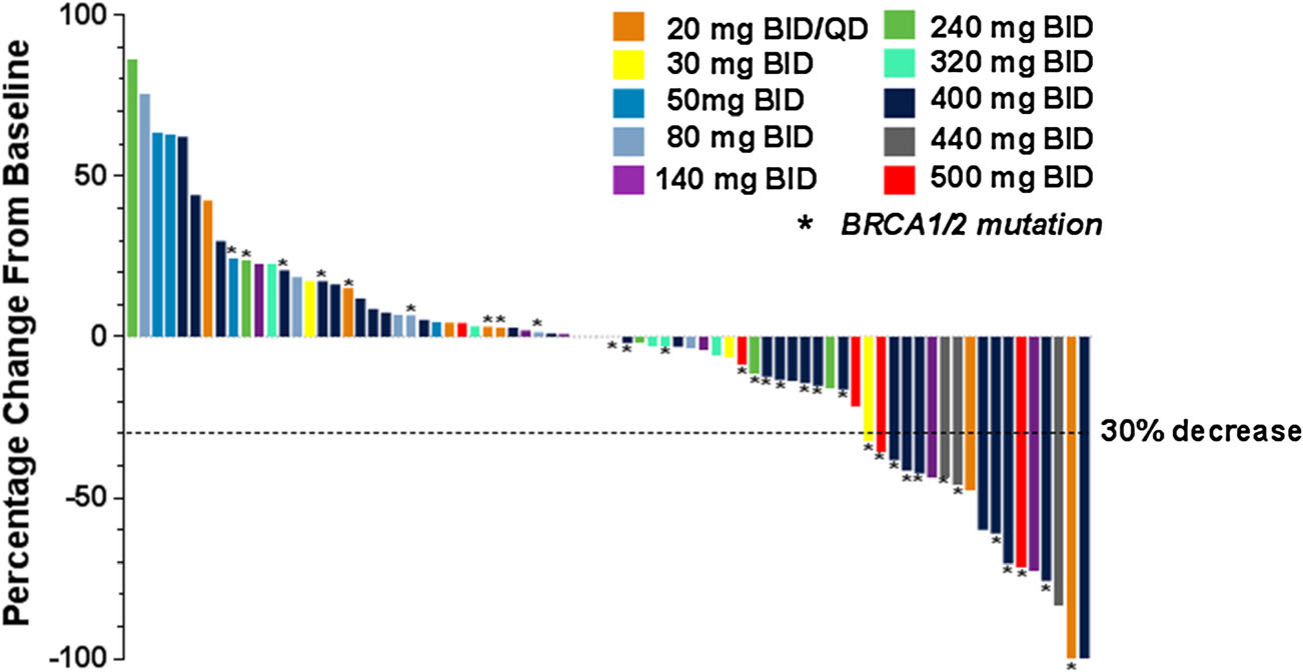
Best percentage change from baseline in tumor size by ABT-767 dose in all patients. Abbreviations: *BID* twice daily; *QD* once daily. Germline *BRCA* status was provided by the investigators

The best percentage change from baseline in tumor size by ABT-767 dose is shown in Fig. 3. A ≥ 30% reduction from baseline in tumor size was seen in 19 of 76 patients who had a post-baseline measurement.

The 6-month PFS rate was 33% (95% CI: 23–42%) for all patients, and 32% (95% CI: 22–42%) for patients with ovarian cancer. The median PFS was 3.8 months (95% CI: 2.8–5.2 months) for all patients, 3.7 months (95% CI: 2.7–4.7 months) for patients with ovarian cancer, and 5.6 months (95% CI: 1.8–7.7 months) for patients with other types of primary cancer.

### Biomarker analysis

Somatic *BRCA* mutation status and HRD status were determined for 60 patients with ovarian cancer for whom tissue was submitted. Thirty-four patients had ovarian tumors that were HRD positive; of these, 26 had deleterious *BRCA* mutations. Of the 34 HRD positive patients, 16 (47%) were responders (7 PR, 9 CR) per RECIST 1.1 and/or CA-125 criteria; all 16 responders had prior platinum and 2 were platinum resistant. Among the HRD positive patients who had a deleterious somatic *BRCA* mutation, 14/26 (54%) were responders (7 PR, 7 CR) per RECIST 1.1 and/or CA-125 criteria (Table 3). Among the 8 patients who were HRD positive but had no deleterious *BRCA* mutation, 2 were responders by RECIST 1.1 and/or CA-125 criteria. Both of these patients were partially platinum sensitive, received ABT-767 at 400 mg BID and had a CR. Among patients determined to be HRD negative, there were no responders per RECIST 1.1 or CA-125 criteria. Among HRD positive patients with ovarian cancer, responses were generally more frequent in patients with fewer prior therapies (Supplemental Table 2)**.**

**Table 3:** Tumor response by RECIST 1.1 and/or CA-125 by HRD and somatic *BRCA1/2* mutation status in patients with ovarian cancer

n (%)	Complete Response	Partial Response	Non-Responder
HRD positive (N = 34)	9 (26%)	7 (21%)	18 (53%)
*BRCA1/2* mutation (*N* = 26)	7 (27%)	7 (27%)	12 (46%)
*BRCA1/2* wild-type (*N* = 8)	2 (25%)	0	6 (75%)
HRD negative (N = 26)	0	0	26 (100%)
HRD undetermined (*N* = 7)	1 (14%)	0	6 (86%)

Abbreviations: *HRD* homologous recombination deficiency

PFS was significantly longer in HRD positive patients with ovarian cancer (median PFS 6.7 months; *n* = 34) compared with HRD negative patients (median PFS 1.8 months; *n* = 26) (Log rank *p* < 0.0001) (Supplemental Fig. 2).

### Predictors of response

In univariate analysis, platinum sensitivity (compared to platinum resistant population) was a significant covariate (*p* < 0.01) affecting best tumor response by RECIST, whereas ABT-767 dose and *BRCA* mutational status (germline compared to non-germline) showed a trend toward significance (*p* < 0.1) (Supplemental Fig. 3). In multivariate analysis, platinum sensitivity was a statistically significant covariate affecting the best tumor response by RECIST (*p* < 0.01). Both univariate and multivariate analyses revealed that PFS is significantly affected by *BRCA* mutational status (germline compared to non-germline; *p* < 0.05) and by platinum sensitivity (platinum sensitive compared to platinum resistant population; *p* < 0.05) (Supplemental Fig. 3 B-C).

## Discussion

This phase 1 study evaluated ABT-767 in patients with ovarian cancer or *BRCA* mutations. ABT-767 had an acceptable safety profile up to the established RP2D of 400 mg BID. Anemia was the most common grade 3/4 TEAE; onset of anemia was monitorable and was generally manageable with standard supportive care and dose reduction. Anemia has been frequently reported with other PARP inhibitors [4, 7, 8]. The half-life of ABT-767 was approximately 2 h and renal clearance was a significant pathway for ABT-767 elimination. The exposure to ABT-767 increased approximately dose-proportionally from 20 mg to 500 mg. Food had no significant effect on ABT-767 oral bioavailability up to 400 mg dose. The data suggest that ABT-767 has single-agent activity in patients with tumors with *BRCA* mutations or high-grade serous ovarian cancer, with tumor responses of 21% (17/80 patients) in all patients per RECIST 1.1 criteria, and 30% (24/80 patients) in patients with ovarian cancer per CA-125 and/or RECIST 1.1 criteria.

Biomarker analyses indicate ABT-767 sensitivity among HRD positive patients with ovarian cancer. PFS was significantly prolonged in patients who were HRD positive, and observed RECIST and/or CA-125 responses were generally restricted to HRD positive patients. Responses were generally more common among HRD positive patients who had a somatic *BRCA* mutation compared to those who did not; however, the sample size of HRD positive *BRCA* wild-type patients was small at only 8 patients. Patient selection with a functional HRD test [16] or RAD51 assay [18] may be useful for identifying patients likely to respond. The biomarker analyses are limited by the collection of tissue in a subset of patients, and the inclusion of archived tissue that may have been from the time of diagnosis in patients who received multiple prior lines of therapy. It was observed that patients were generally less likely to respond with increasing number of prior lines of therapy. Mechanisms of resistance and possible *BRCA1/2* reversion mutations were not evaluated in this study. Univariate and multivariate analyses showed that PFS is significantly affected by *BRCA* mutation and platinum sensitivity, further delineating patient populations that may benefit from therapy.

In this phase 1 study of ABT-767, responses were observed in a refractory, heterogeneous patient population. Patients with *BRCA* mutations, HRD positivity, and platinum sensitivity were more sensitive to treatment, supporting that these populations are suitable candidates for PARP inhibitor therapy.

## Electronic supplementary material

ESM 1 (DOCX 214 kb)
