# Supplementary material for: A phase 1 study of PARP-inhibitor ABT-767 in advanced solid tumors with BRCA1/2 mutations and high-grade serous ovarian, fallopian tube, or primary peritoneal cancer
Source: Invest New Drugs. 2018 Jan 8;36(5):828–35. doi: 10.1007/s10637-017-0551-z (PMC6153550; doi:10.1007/s10637-017-0551-z)
Supplement: Supplementary file 1 — (DOCX 214 kb) [file 10637_2017_551_MOESM1_ESM.docx]

**A Phase 1 Study of PARP-inhibitor ABT-767 in Advanced Solid Tumors With BRCA1/2 Mutations and High-Grade Serous Ovarian, Fallopian Tube, or Primary Peritoneal Cancer**

**Journal name:** *Investigational New Drugs*

**Authors:** Diane A.J. van der Biessen, Jourik A. Gietema, Maja J.A. de Jonge, Ingrid M.E. Desar, Martha W. den Hollander, Matthew Dudley, Martin Dunbar, Robert Hetman, Camille Serpenti, Hao Xiong, Rajendar K. Mittapalli, Kirsten M. Timms, Peter Ansell, Christine K. Ratajczak, Stacie Peacock Shepherd, Carla M.L. van Herpen

**Corresponding Author:**

Carla M.L. van Herpen

Radboud University Medical Center,

Nijmegen, The Netherlands

Email: carla.vanherpen@radboudumc.nl

**SUPPLEMENTARY MATERIAL INCLUDED:**

Supplemental Figures 1, 2, and 3; Supplemental Tables 1 and 2.

**SUPPLEMENTARY MATERIAL**

**RESULTS**

**Supplemental Figure 1.** Mean hemoglobin level from screening visit to Cycle 3 Day 1


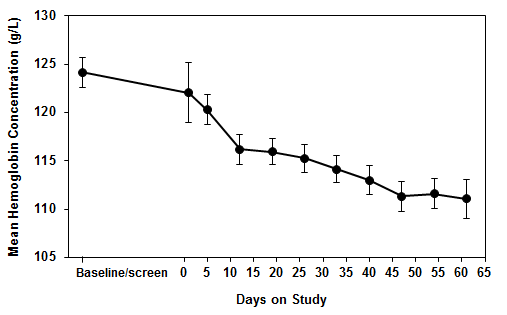


Mean ± standard error included all patients' laboratory data that was collected from screening visit to Cycle 3 Day 1.

**Supplemental Table 1.** Geometric mean (mean, % CV) pharmacokinetic parameters of ABT-767 after the morning dose on Day 1 of Cycle 1

| ABT-767  Dose Cohort | N | C_max_ (μg/mL) | T_max_^b^ (h) | AUC_10_ (μg•h/mL) | AUC_∞_ (μg•h/mL) | t_1/2_^c^ (h) | CL/F (L/h) | | V_dβ_/F (L) |
| --- | --- | --- | --- | --- | --- | --- | --- | --- | --- |
| 20 mg QD | 3 | 0.752 (0.770, 26) | 2.0 (2.0 - 4.0) | 3.37 (3.45, 27) | 3.90 (3.96, 24) | 4.2 ± 0.5 | 5.13 (5.21, 21) | | 31.5 (32.5, 29) |
| 20 mg BID | 5 | 0.592 (0.624, 32) | 2.0 (1.5 - 4.0) | 2.31 (2.44, 35) | 2.44 (2.59, 37) | 2.1 ± 0.4 | 8.20 (8.74, 40) | | 24.9 (26.2, 36) |
| 30 mg BID | 3 | 1.07 (1.07, 11) | 2.0 (1.5 - 2.0) | 4.42 (4.67, 43) | 4.75 (5.18, 53) | 2.0 ± 0.7 | 6.31 (6.80, 42) | | 19.3 (19.3, 8) |
| 50 mg BID | 6 | 1.53 (1.61, 32) | 1.8 (1.5 - 4.0) | 6.54 (6.78, 29) | 7.05 (7.37, 31) | 1.9 ± 0.4 | 7.09 (7.45, 37) | | 19.6 (19.9, 21) |
| 80 mg BID | 8 | 3.11 (3.18, 23) | 2.0 (1.0 - 4.0) | 13.9 (14.4, 30) | 15.1 (15.9, 33) | 2.3 ± 0.4 | 5.28 (5.54, 32) | | 17.4 (17.7, 20) |
| 140 mg BID | 6 | 4.00 (4.07, 20) | 1.5 (1.0 - 2.0) | 16.3 (16.5, 19) | 17.2 (17.5, 22) | 2.1 ± 0.5 | 8.15 (8.31, 22) | | 24.8 (25.0, 12) |
| 240 mg BID | 5 | 5.42 (5.66, 36) | 2.0 (1.5 - 4.9) | 22.8 (23.9, 39) | 25.6 (26.9, 41) | 1.7 ± 0.3 | 9.39 (9.82, 33) | | 23.6 (23.8, 17) |
| 320 mg BID | 6 | 5.61 (5.67, 16) | 2.0 (2.0 - 2.0) | 30.4 (30.5, 9.0) | 32.7 (32.8, 9.0) | 1.9 ± 0.3 | 9.79 (9.82, 8.0) | | 27.7 (28.0, 18) |
| 400 mg BID  Dose Escalation | 6 | 9.40 (9.92, 33) | 2.0 (1.5 - 4.0) | 41.6 (42.9, 28) | 47.7 (49.1, 27) | 1.9 ± 0.5 | 8.39 (8.65, 27) | | 23.9 (25.1, 35) |
| 400 mg BID  Expanded Safety^a^ | 24 | 8.90 (9.74, 49) | 1.5 (1.0 - 10.0) | 38.3 (41.1, 42) | 40.1 (43.2, 43) | 2.1 ± 0.8 | 9.97 (10.6, 33) | | 31.0 (33.8, 47) |
| 440 mg BID | 5 | 13.6 (14.4, 37) | 1.5 (0.5 - 6.0) | 62.6 (66.5, 39) | 64.6 (70.2, 51) | 2.6 ± 0.8 | | 6.82 (7.32, 39) | 26.2 (26.8, 24) |
| 500 mg BID | 6 | 11.8 (13.8, 64) | 1.8 (1.5 - 4.0) | 56.7 (65.3, 60) | 60.5 (70.2, 61) | 1.9 ± 0.5 | | 8.26 (9.40, 50) | 1. (26.8, 44) |

^a^ All other cohorts were dose escalation cohorts.

^b^ Median (minimum - maximum).

^c^ Harmonic mean ± pseudo-standard deviation.

Abbreviations: AUC_10_, area under the plasma concentration–time curve from time 0 to 10 hours; AUC_∞_, total area under the concentration–time curve; BID, twice daily; CL/F, clearance as a function of bioavailability; C_max_, maximum observed plasma concentration; QD, once daily; t_1/2_, elimination half-life; T_max_, time to maximum observed plasma concentration; V_dβ_/F, volume of distribution as a function of bioavailability.

**Supplemental Table 2.** Tumor response by RECIST 1.1 and/or CA-125 by number of prior therapies among HRD positive patients with ovarian cancer

| **Number of prior regimens** | **Non-responders**  **n/N (%)** | **Partial Response**  **or Complete Response**  **n/N (%)** |
| --- | --- | --- |
| 1 | 1/5 (20%) | 4/5 (80%) |
| 2 | 4/11 (36%) | 7/11 (64%) |
| 3 | 3/7 (43%) | 4/7 (57%) |
| 4+ | 10/11 (91%) | 1/11 (9%) |

Abbreviations: *HRD* homologous recombination deficiency

**Supplemental Figure 2.** Progression-free survival by HRD status in patients with ovarian cancer.

**
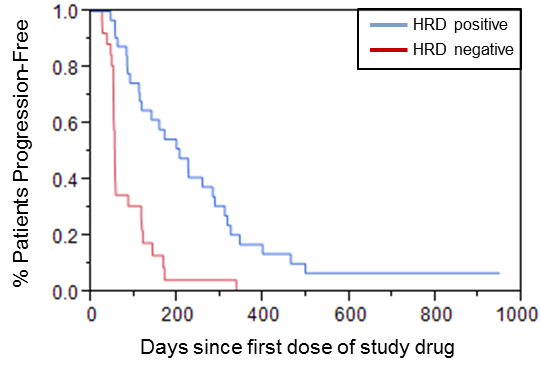
**

Abbreviations: *HRD* homologous recombination deficiency; *PFS* progression-free survival

Legend indicates HRD status. Median PFS was 6.7 months for HRD positive patients and 1.8 months for HRD negative patients (log rank *P*<0. 0001).

**Supplemental Figure 3.** Predicted probability of best tumor response (CR or PR) vs. ABT-767 dose (A) and analysis of progression-free survival based on *BRCA* mutation (B) and platinum sensitivity (C)

**A**

**
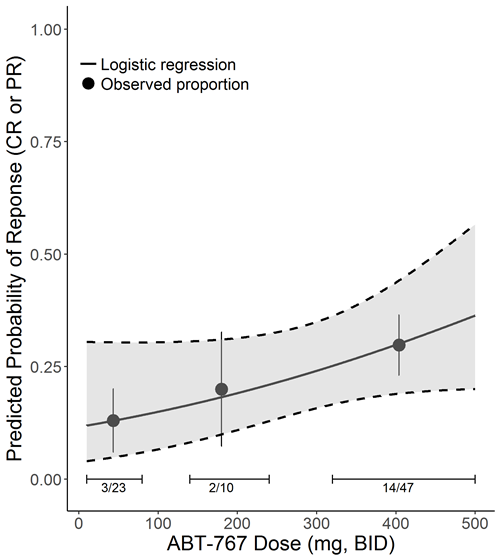
**

**B**

**
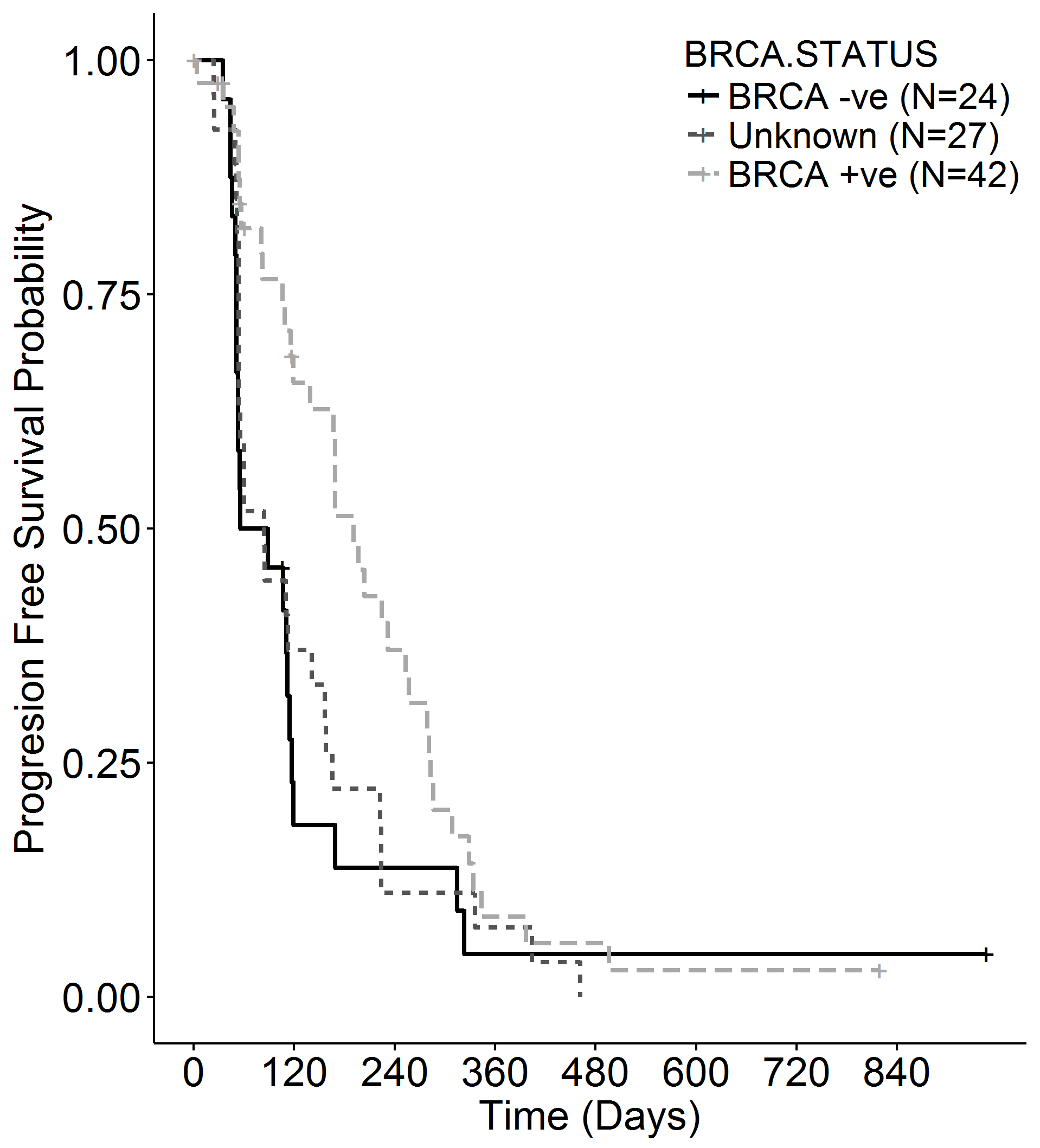
**

**C**


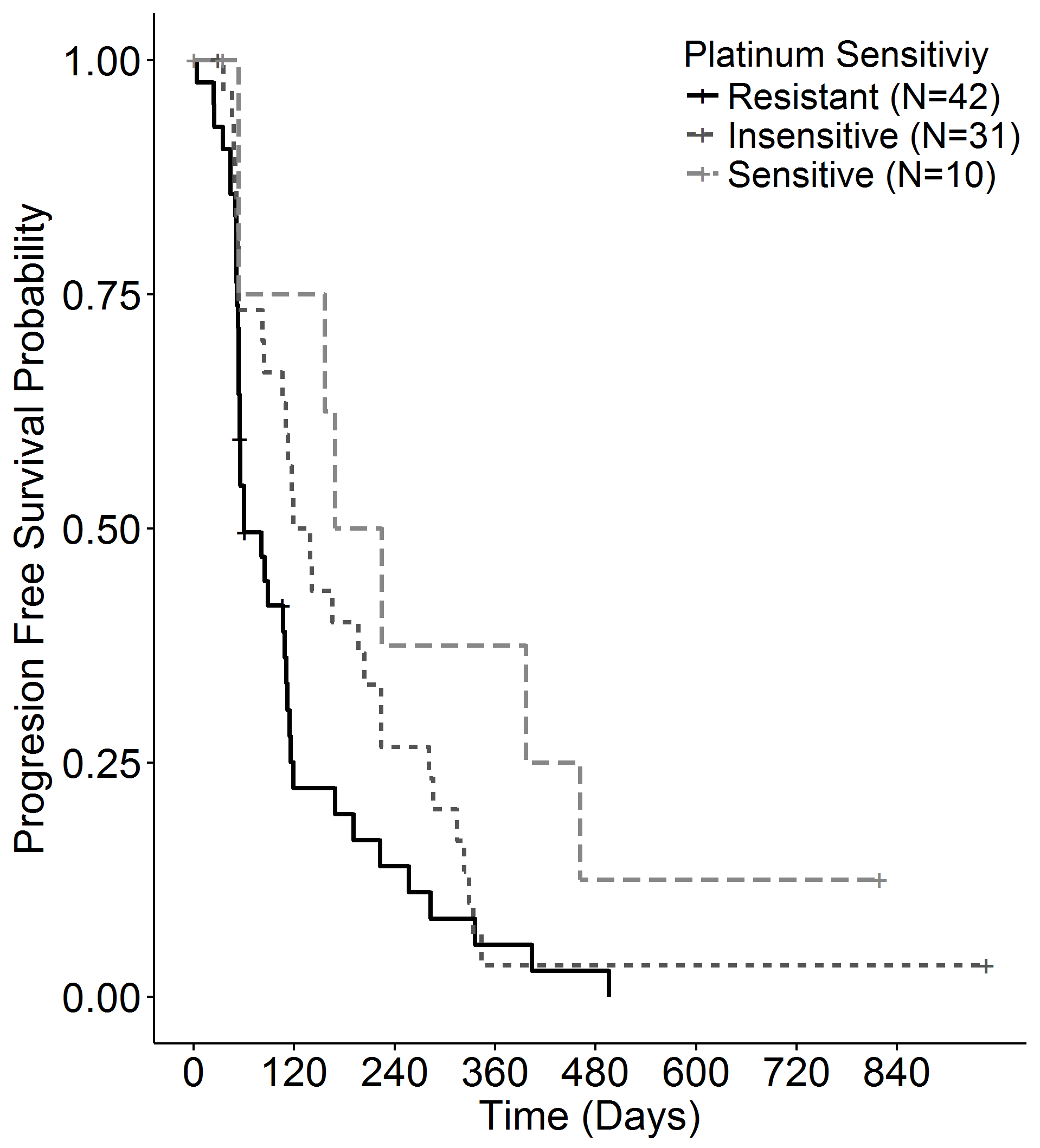


Abbreviations: *BID* twice daily; *CI* confidence interval; *CR* complete response; *PR* partial response

*Part A:* Shaded area between the dashed lines indicates the predicted 95% CI; points with vertical bars indicate the observed proportions with 95% binomial CI at the observed mean ABT-767 dose. Horizontal lines parallel to the x-axis indicate the dose range bins for low (20–80 mg), medium (140–240 mg), and high (320–500 mg) doses. The number of patients with best tumor responses (CR or PR) relative to the total number in that dose bin is shown below the lines.

*Part B:* +ve, patients with germline *BRCA* mutation; -ve, patients without germline *BRCA* mutation.

*Part C:* Platinum sensitivity categories (based on time to progression on platinum therapy) were: resistant 0–6 months; insensitive 6–12 months; sensitive >12 months.
